# FGF10 ameliorates lipopolysaccharide-induced acute lung injury in mice *via* the BMP4-autophagy pathway

**DOI:** 10.3389/fphar.2022.1019755

**Published:** 2022-12-22

**Authors:** Xiaoxia Kong, Liling Lu, Daopeng Lin, Lei Chong, Shunhang Wen, Yaokai Shi, Lidan Lin, Liqin Zhou, Hongyu Zhang, Hailin Zhang

**Affiliations:** ^1^ School of Basic Medical Sciences, Institute of Hypoxia Research, Wenzhou Medical University, Wenzhou, Zhejiang, China; ^2^ Department of Children’s Respiration, The Second Affiliated Hospital & Yuying Children’s Hospital, Wenzhou Medical University, Wenzhou, Zhejiang, China; ^3^ Department of Ultrasound, Children’s Hospital, School of Medicine, Zhejiang University, Hangzhou, Zhejiang, China; ^4^ Department of Nephrology, Affiliated Cangnan Hospital, Wenzhou Medical University, Cangnan, Zhejiang, China; ^5^ Department of Pharmacy, Zhuji People’s Hospital, The Affiliated Hospital of Wenzhou Medical University, Shaoxing, Zhejiang, China; ^6^ School of Pharmaceutical Sciences, Cixi Biomedical Research Institute, Wenzhou Medical University, Wenzhou, Zhejiang, China

**Keywords:** acute lung injury, FGF10, BMP4, autophagy, AECs

## Abstract

**Introduction:** Damage to alveolar epithelial cells caused by uncontrolled inflammation is considered to be the main pathophysiological change in acute lung injury. FGF10 plays an important role as a fibroblast growth factor in lung development and lung diseases, but its protective effect against acute lung injury is unclear. Therefore, this study aimed to investigate protective effect and mechanism of FGF10 on acute lung injury in mice.

**Methods:** ALI was induced by intratracheal injection of LPS into 57BL/6J mice. Six hours later, lung bronchoalveolar lavage fluid (BALF) was acquired to analyse cells, protein and the determination of pro-inflammatory factor levels, and lung issues were collected for histologic examination and wet/dry (W/D) weight ratio analysis and blot analysis of protein expression.

**Results:** We found that FGF10 can prevent the release of IL-6, TNF-α, and IL-1β, increase the expression of BMP4 and autophagy pathway, promote the regeneration of alveolar epithelial type Ⅱ cells, and improve acute lung injury. BMP4 gene knockdown decreased the protective effect of FGF10 on the lung tissue of mice. However, the activation of autophagy was reduced after BMP4 inhibition by Noggin. Additionally, the inhibition of autophagy by 3-MA also lowered the protective effect of FGF10 on alveolar epithelial cells induced by LPS.

**Conclusions:** These data suggest that the protective effect of FGF10 is related to the activation of autophagy and regeneration of alveolar epithelial cells in an LPS-induced ALI model, and that the activation of autophagy may depend on the increase in BMP4 expression.

## Introduction

Acute lung injury (ALI), a critical clinical condition with a high mortality rate, is characterized by an inflammatory reaction in the course of severe infection, shock, trauma, and other non-cardiogenic diseases that exacerbates damage to the pulmonary capillaries and alveolar epithelial cells (AECs). This results in diffuse interstitial and alveolar edema, eventually leading to acute hypoxic respiratory failure or insufficiency (Burnham et al., 2012; Högner et al., 2013). Damage to AECs caused by uncontrolled inflammation is considered to be the main pathophysiological change in acute lung injury. Therefore, protecting against the damage of AECs and maintaining the integrity of pulmonary epithelial cell function are one of the strategies for the treatment of ALI.

Fibroblast growth factor 10 (FGF10) is a typical FGF composed of 215 amino acids (Itoh, 2016) that binds to receptors *via* paracrine or autocrine pathways, acting on cofactor heparin sulfate and binding to fibroblast growth factor receptor 2 (FGFR2b) receptors, which play an important role in adult development and tissue homeostasis (McCormack et al., 2013; Prince, 2018). It leads to the regulation of organ branching and cell proliferation, differentiation and migration, wound healing and tissue repair, maintenance of stem cell compartments, and invasion and proliferation of cancer cells during development (Itoh, 2016). Studies have shown that FGF10 plays an important role in lung development and disease. It can regulate lung branching development *via* the FGF10-FGFR2b-sprouty pathway (Unbekandt et al., 2008). In a model of acute lung injury induced by bleomycin, bronchial epithelial cells were prompted to produce basal cells by FGF10 signaling, which also encourages the regeneration of AECs to improve acute lung injury (Yuan et al., 2019). However, the protective mechanism of FGF10 in ALI is not fully understood.

Autophagy is a dynamic process in the turnover of organelles and proteins through a lysosome-associated degradation process (Klionsky et al., 2021). It has long been considered a double-edged sword, as its degradation products provide energy and material sources for cell regeneration; however, excessive activation of autophagy can lead to cell damage (Mizumura et al., 2014; Sui et al., 2017; Pickles et al., 2018). There is evidence that autophagy is upregulated in most lung diseases initially as a pre-survival mechanism responsible for clearing damaged organelles and proteins (Racanelli et al., 2018). Autophagy can alleviate the injury of AECs by regulating the release of inflammatory mediators; and the disruption of autophagy in AECs is essential for the development of pulmonary fibrosis (Tang et al., 2017). Additionally, FGF10 has been found to protect against renal ischemia-reperfusion injury by regulating autophagy and controlling the differentiation of cardiac stem cells by autophagy regulation in other diseases (Zhang et al., 2012; Tan et al., 2018). However, it is unclear whether there is a link between FGF10 and autophagy in acute lung injury. Bone morphogenetic protein 4 (BMP4) belongs to the transforming growth factor superfamily β (TGF-β) superfamily and is present in many types of cells (Salazar et al., 2016). During lung development, BMP4 is a downstream signaling molecule of FGF10 (Yuan et al., 2018). The presence of BMP4 in ATII cells contributes to alveoli stability and regulates the growth and differentiation of epithelial cells (McCormack et al., 2013). Meanwhile, increased expression of BMP4 in pseudostratified columnar epithelial cells reduces inflammation (Li et al., 2019). It has also been reported that BMP4 is located upstream of autophagy, and it can promote the proliferation of hepatoma cells (Deng et al., 2018) as well as increase the resistance to leukemic chemotherapy drugs by activating autophagy, subsequently inhibiting apoptosis (Zhao et al., 2013).

This study evaluated the protective effect of FGF10 on lipopolysaccharide (LPS)-induced acute lung injury in mice and ACE cells. We used a non-specific PI3 kinase inhibitor, 3-MA, to further explore the relationship between FGF10 and autophagy in acute lung injury. In addition, Noggin, a BMP4 inhibitor, and BMP4 knockdown mice were used to explore whether the protective effect of FGF10 was associated with BMP4. Our results showed that FGF10 improved acute lung injury and could be associated with autophagy activated by BMP4.

## Materials and methods

### Animals

Adult male C57BL/6J mice (20–25 g, 6–8 weeks old) were purchased from Vital River Laboratory Animal Technology Co., Ltd (Beijing, China). The mice were placed in an environment with free access to food and water during 12-h dark/light cycles. The animals were randomly divided into four groups (n = 8–10/group): control, LPS, FGF10 + LPS, and FGF10. The mice were anesthetized with chloral hydrate. FGF-10, saline, and LPS were injected into the trachea using an 18G catheter attached to a 1-ml syringe. For the LPS and FGF-10 groups, LPS (Sigma-Aldrich, L6386) and FGF-10 at a dose of 5 mg/kg were instilled into the lungs of mice through a catheter. The control animals received an equal volume of PBS. For the mice in the FGF10 + LPS group, FGF-10 was instilled into their lungs for 24 h, followed by LPS treatment for 6 h. Finally, the mice were anesthetized with chloral hydrate for bronchoalveolar lavage fluid (BALF) and lung tissue collection. To test whether BMP4 knockdown resulted in an ameliorative effect on FGF10-protected ALI. The mice were randomly divided into two groups: 1) scrambled control and 2) BMP4 shRNA. For AAV-BMP4 shRNA or BMP4 scramble control treatment, the tail veins of mice were injected with AAV- BMP4 shRNA or the BMP4 scramble control once at a dose of 2.0 × 10^11^ vg for 3 weeks, and the mice were intratracheally administered FGF10 and/or LPS as described above. All animal studies were conducted under the supervision of the Institutional Animal Care and Use Committee of Wenzhou Medical University.

### Hematoxylin and eosin staining

After collection, lung tissues were fixed with 4% paraformaldehyde. These were then paraffin-embedded and prepared into 5-μm slices. Subsequently, these sections were de-paraffinized, rehydrated, H&E-stained, and analyzed using a light microscope.

### Bronchoalveolar lavage fluid collection

For BALF collection, 1 ml of PBS was gradually injected into the lungs and pumped out three times; the alveolar lavage fluid was collected using a syringe. After collection, the BALF samples were centrifuged at 1500 rpm for 10 min.

### Wet/dry weight ratio

The lung tissue was removed and the wet weight was measured. The lungs were then placed in an oven at 60°C for 48 h to determine the dry weight. Then, the wet/dry weight ratios were calculated.

### Enzyme-linked immunosorbent assay

The levels of IL-6, TNF-α, and IL-1β in the BALF and total protein were quantified using the indicated ELISA kits (DAKEWE, 1210122, 1210602, 1217202). Simply, the sample and various concentrations of standard cytokines were added to the wells and incubated with biotinylated antibodies at 37°C for 90 min. We followed all procedures as described previously (Zhang et al., 2018).

### Western blotting

Lung tissues were homogenized, and total proteins were extracted using tissue lysis buffer supplemented with phenylmethanesulfonyl fluoride. In the *in vitro* experiment, the total proteins of AECs were extracted using a cell lysis buffer. For the quantification of protein content, a BCA kit (Beyotime, P0010S) was used. Denatured proteins were added to denaturing gels and separated by SDS-PAGE (Mini-PROTEAN Tetra electrophoresis system, Bio-Rad). The separated proteins were then transferred onto polyvinylidene fluoride membranes, and the membranes were incubated with primary antibodies. Primary antibodies against LC3B (Abcam, ab128025,1:1000), P62 (Abcam, ab56416,1:1000), BMP4 (Abcam, ab39973,1:800), SPC (Abcam, ab90716,1:900), FGF10 (Abcam, ab71794,1:1000), FGFR2 (Abcam, ab10648,1:2000) and GAPDH (Bioworld, AP0063,1:20000) were used at 4°C overnight after blocking with skim milk. The PVDF membranes were incubated with secondary antibodies (BioWorld, goat anti-rabbit, BS13278; goat anti-mouse, BS12478) and visualized by chemiluminescence the following day. Finally, the gray value of each band was measured using ImageJ software.

### Cell culture

Human AECs were purchased from BLUEFBIO (Shanghai, China) and were grown in DMEM, supplemented with 10% fetal bovine serum (Gibco, 10099141) and penicillin/streptomycin (Gibco, 15140122) at 37°C with 5% CO_2_.

### Cell viability measurement

Next, 1×10^4^ cells/well were plated in 100 μL of 96-well plates, and cell viability was detected by the CCK-8 assay (Beyotime, C0038). Briefly, the cells were treated with FGF10 (10 ng/ml) for 1 h before and then exposed to LPS (10 μg/ml) for 24 h. For detecting the role of BMP4 or autophagy in FGF10 protection against LPS-induced lung injury, the inhibitor of BMP4, Noggin (200 ng/ml) or the inhibitor of autophagy, 3-MA (2.5 mmol/L) are added to ACEs before exposure of FGF10 for 30min. Finally, 10 μL of CCK-8 developing solution was added to each well at 37°C for 1 h. The absorbance at 450 nm was measured using a microplate reader.

### Immunofluorescence staining

Cells were seeded on slides in a cell culture chamber and allowed to adhere overnight. The next day, the cells were washed three times with PBS buffer, fixed with 4% paraformaldehyde, cultured with 0.1% Triton x-100 for 15 min, and sealed with 5% BSA for 30 min to block non-specific binding and permeability of the cell membrane. Thereafter, the cells were incubated overnight with the primary antibody at 4°C. The cells were incubated with a secondary antibody (Cell Signaling Technology, 4412S) for 1 h the following day. After three washes with PBS buffer, the nuclei were stained with DAPI (Beyotime, C1005) and viewed under a fluorescence microscope. All staining procedures were performed using the appropriate isotype controls.

### Statistical analysis

The data were expressed as the mean ± SEM from several independent experiments (n ≥ 3). GraphPad Prism 8.0 (San Diego, California, United States) was used for all statistical analyses. The differences between the two groups were evaluated using Student’s t-test. Multiple group comparisons were performed using one-way ANOVA. Differences were considered statistically significant at *p* < 0.05.

## Results

### FGF10 is upregulated in LPS-induced lung injury

**FIGURE 1:**
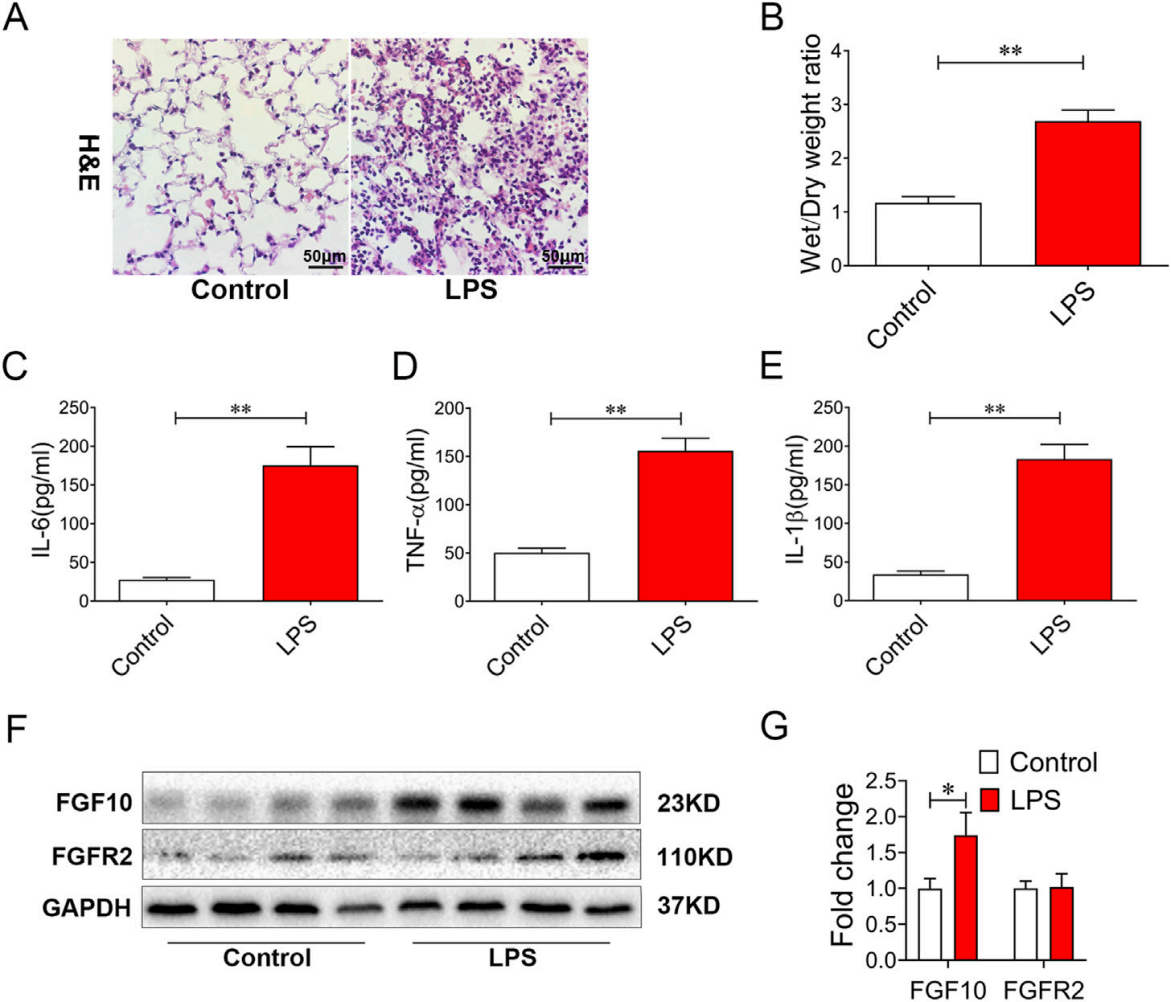
LPS treatment results in alveolar injury and increases FGF10 expression in mice. **(A)** Pathological change in lung tissue was detected by H&E (200×). **(B)** Quantification analysis of wet/dry weight ratio. **(C–E)** The expression of IL-6, TNF-α, and IL-1β in BALF were determined by ELISA. **(F)** The expression of FGF10 and FGFR2B were detected by Western blotting. **(G)** Quantitative analysis of FGF10 and FGFR2B protein. The data were presented as the mean ± SEM, n = 5. *p < 0.05, * *p < 0.01.

Wefirst tested our hypothesis in an LPS-induced ALI mouse model. As anticipated, the lung sections from LPS-treated mice for 6 h displayed marked alveolar damage, structural destruction, and accumulation of fibrotic collagen in the alveolar parenchyma (Figure 1A). Moreover, the ratio of wet/dry weight was increased in LPS-induced lungs (*p* < 0.01) (Figure 1B), and alveolar lavage fluid showed an increase in the inflammatory mediators IL-6, TNF-α, and IL-1β (Figures 1C–E). Finally, we measured the expression of FGF10 and its ligand FGFR2 by Western blotting, and found that the expression of FGF10 was significantly increased in LPS-induced ALI (*p* < 0.05), but the expression of the FGF10 receptor did not significantly change (Figure 1F and 1G). These results suggest that LPS increased the expression of FGF10 in an acute lung injury mouse model.

### FGF10 resists LPS- induced lung injury

**FIGURE 2:**
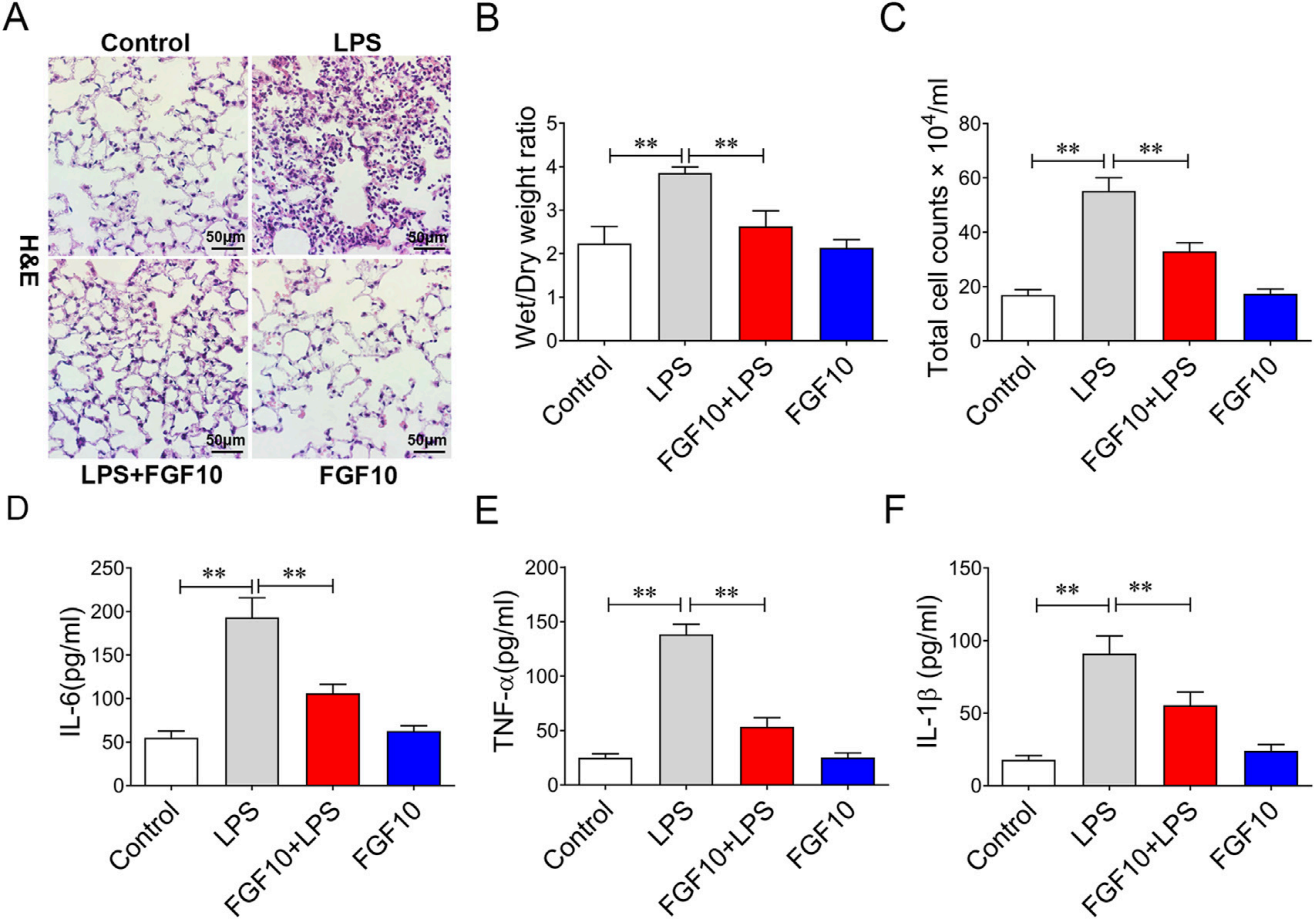
FGF10 attenuates LPS-induced structural damage and inflammatory mediator production in mice. **(A)** Pathological change in lung tissue was detected by H&E (200×). **(B)** Quantitative analysis of wet/dry weight ratio. **(C)** BAL fluid was obtained to differentially count total cells.**(D–F)** The expression of IL-6, TNF-α, and IL-1β in BALF were determined by ELISA. The data were presented as the mean ± SEM, n = 5. *p < 0.05, * *p < 0.01.

To understand the role of FGF10 in acute lung injury, we used FGF10 to treat mice with acute lung injury. H&E staining data showed that there were no abnormal changes in lung histomorphology in the control group and mice treated with 5 mg/kg of FGF10 alone. There was marked infiltration of inflammatory cells into the alveolar spaces and peribronchial wall thickening in the lungs of ALI mice. These histological changes were dramatically alleviated by FGF10 administration (Figure 2A). LPS caused an increase in the wet/dry weight ratio that was also lowered by FGF10 treatment (Figure 2B). Furthermore, to investigate the anti-inflammatory effect of FGF10 in LPS-induced acute lung injury, the number of total cells and some typical inflammatory cytokines in alveolar lavage fluid (BALF) were measured by ELISA. The results showed that LPS markedly increased the total cells in alveolar lavage fluid and levels of IL-6, TNF-α, and IL-1β, which were significantly decreased by FGF10 treatment (Figures 2C–F). These results confirm that FGF10 improves LPS-induced acute lung injury.

### FGF10 elevated autophagic levels in LPS-induced acute lung injury

**FIGURE 3:**
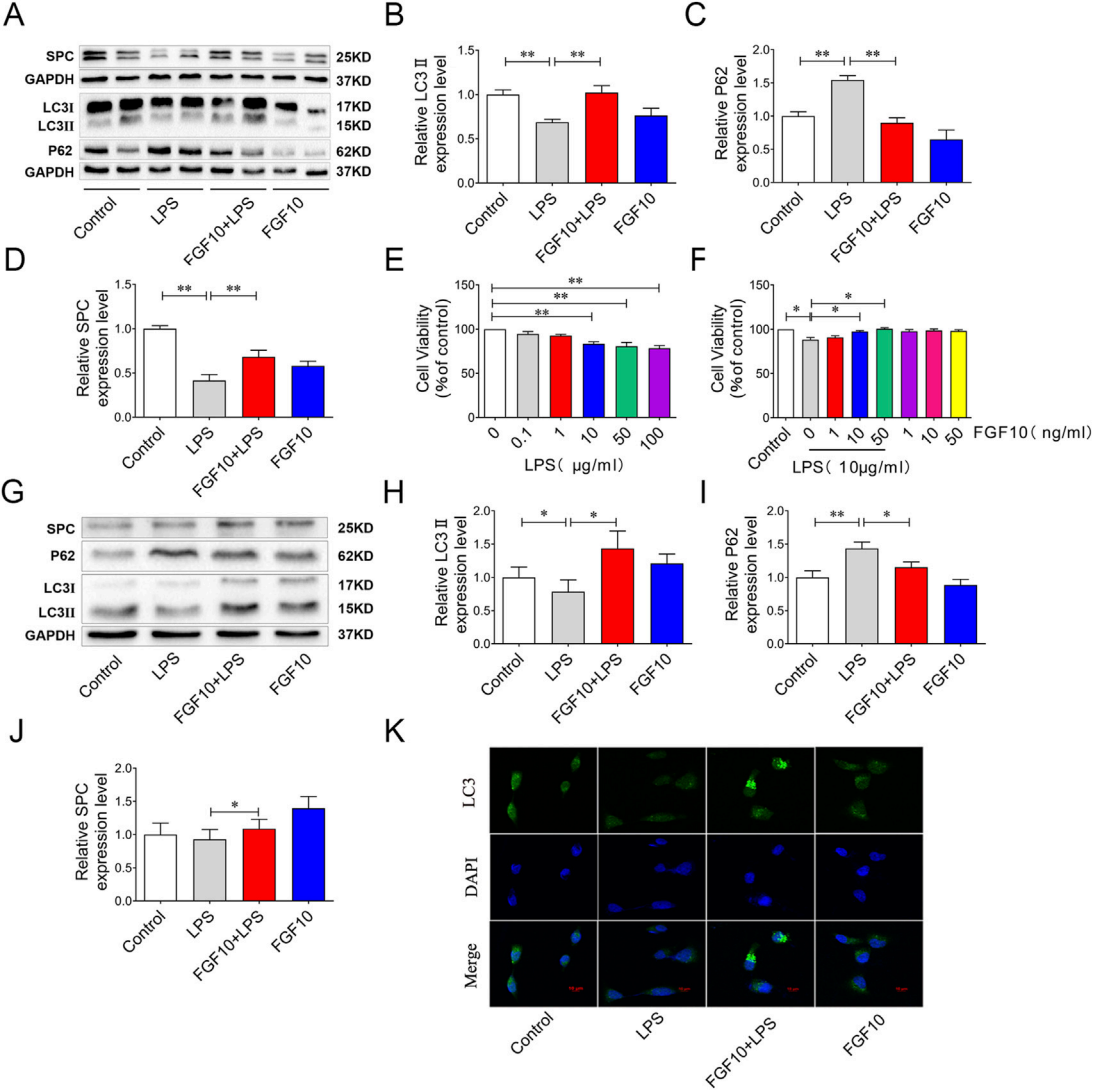
FGF10 promotes autophagy and the regeneration of alveolar epithelial cells in LPS-induced lung injury. **(A)** The expression of LC3, P62, and SPC in mice was detected by Western blotting. **(B–D)** Quantitative analyses of LC3, P62, and SPC protein. **(E)** The viability of AECs with different concentrations of LPS administration was detected by CCK-8. **(F)** After exposure to LPS and FGF10, the viability of AECs were detected by CCK-8. **(G)** The expression of LC3, P62, and SPC in AECs was detected by Western blotting. **(H–J)** Quantitative analysis of LC3, P62, and SPC protein. **(K)** The expression of LC3 (green) of AECs was analyzed by immunostaining (magnification ×400). The data were presented as the mean ± SEM, n = 3–5. *p < 0.05, * *p < 0.01.

Studies have shown that autophagy has varying degrees of protection or injurious effects in most lung diseases (Liu et al., 2017; Wei et al., 2020). To investigate whether the protective mechanism of FGF10 is related to autophagy, we measured autophagic flux in our experiment. LC3 is one of the most common markers of autophagy, and the transformation of LC3I to LC3II indicates its induction. Western blotting analysis showed that the ratio of LC3II/LC3I was lower and the expression of P62 was higher in the LPS group than in the control group, suggesting that autophagy flux was inhibited by LPS. However, treatment with FGF10 significantly increased the ratio of LC3II/LC3I and decreased the expression of P62 (Figures 3A–C), indicating that FGF10 promoted autophagy in ALI models. Additionally, compared with the control group, surfactant-associated protein C (SPC), a marker of alveolar type II cells, was decreased in LPS-induced lung injury, but was significantly increased by FGF10 treatment (Figure 3A–D). Meanwhile, the effect of FGF10 was further determined on alveolar epithelial cells. Cells were treated with different concentrations (0.1, 1, 10, 50, 100 μg/ml) of LPS for 24h, the cell viability was detected with CCK8 assays. The results showed that the cell viability was significantly decreased, when the concentration of LPS was greater than 10 μg/ml (Figure 3E). Furthermore, different concentrations of FGF10 were added to the cells. CCK-8 assays showed that compared with the control group, different concentrations (1, 10, 50 ng/ml) of FGF10 had no significant effect on cell viability, but administration of FGF10 at or above 10 ng/ml for 1 h in advance could significantly alleviate the LPS-induced decrease in cell viability (Figure 3F). Therefore, we selected 10 ng/ml of FGF10 for subsequent experiments. Subsequently, the change in autophagic flux was detected by Western blotting analysis, which showed that LPS markedly decreased the ratio of LC3II/LC3I and increased the expression of P62, though reversed by FGF10 (Figures 3G–I). Meanwhile, the expression of SPC was also detected by Western blotting, and the results were similar to those of the *in vivo* experiment (Figure 3G–J). Immunofluorescence analysis further detected autophagy, and the results showed that compared with the LPS group, the formation of LC3 puncta markedly increased after treatment with FGF10 (Figure 3K), which was consistent with the results of *in vivo* experiments. These results indicate that FGF10 alleviates LPS-induced ALI and may be related to the activation of autophagy.

### FGF10 promotes BMP4 expression in LPS-induced lung injury model

**FIGURE 4:**
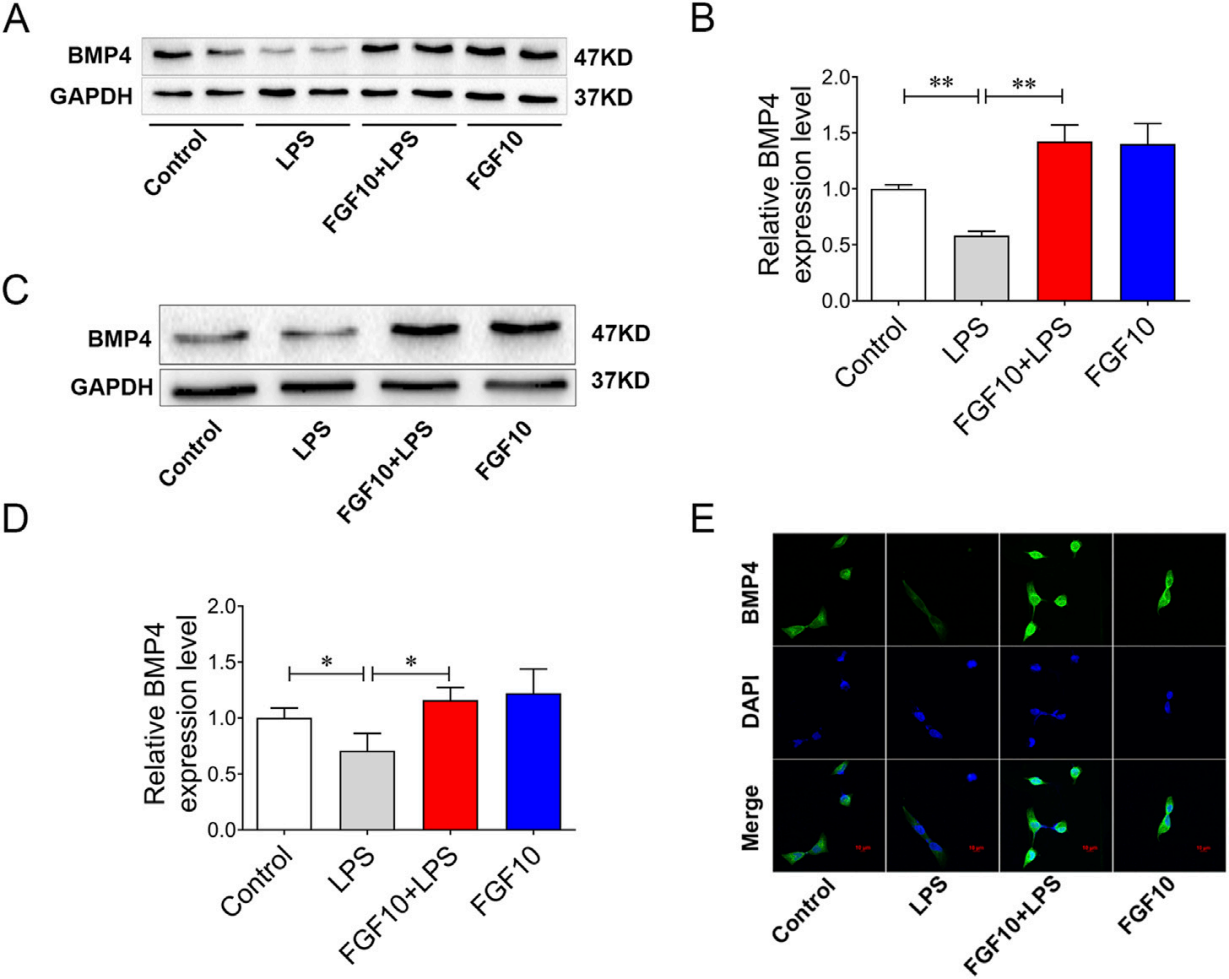
FGF10 promotes the expression of BMP4. **(A)** The expression of BMP4 in mice was detected by Western blotting. **(B)** Quantitative analysis of BMP4 protein. **(C)** The expression of BMP4 in AECs was detected by Western blotting. **(D)** Quantitative analysis of BMP4 protein. **(E)** BMP4 expression (green) of AECs was analyzed by immunostaining (magnification ×400). The data were presented as the mean ± SEM, n = 3–5. *p < 0.05, * *p < 0.01.

BMP, a member of the TGF-β, is one of the downstream signaling factors of FGF10 (Kahata et al., 2018; Yuan et al., 2018). To further explore whether the protective effect of FGF10 is related to BMP4, we examined the expression of BMP4 by Western blotting analysis and found that the expression of BMP4 was significantly decreased in ALI mice; however, this could be reversed by the tracheal injection of FGF10 (Figures 4A, B). Meanwhile, expression of BMP4 was further confirmed in an LPS-induced AEC injury model, and western blotting showed that the expression of BMP4 decreased following LPS injury; similarly, this was reversed by FGF10 treatment (Figures 4C, D). Immunofluorescence showed that the expression of BMP4 in FGF10+ LPS-treated cells was higher than that in LPS-treated cells (Figure 4E). These results indicate that the protective effect of FGF10 could be related to the promotion of BMP4 expression.

### BMP4-knockdown attenuated the protective effect of FGF10 on ALI mice and autophagy

**FIGURE 5:**
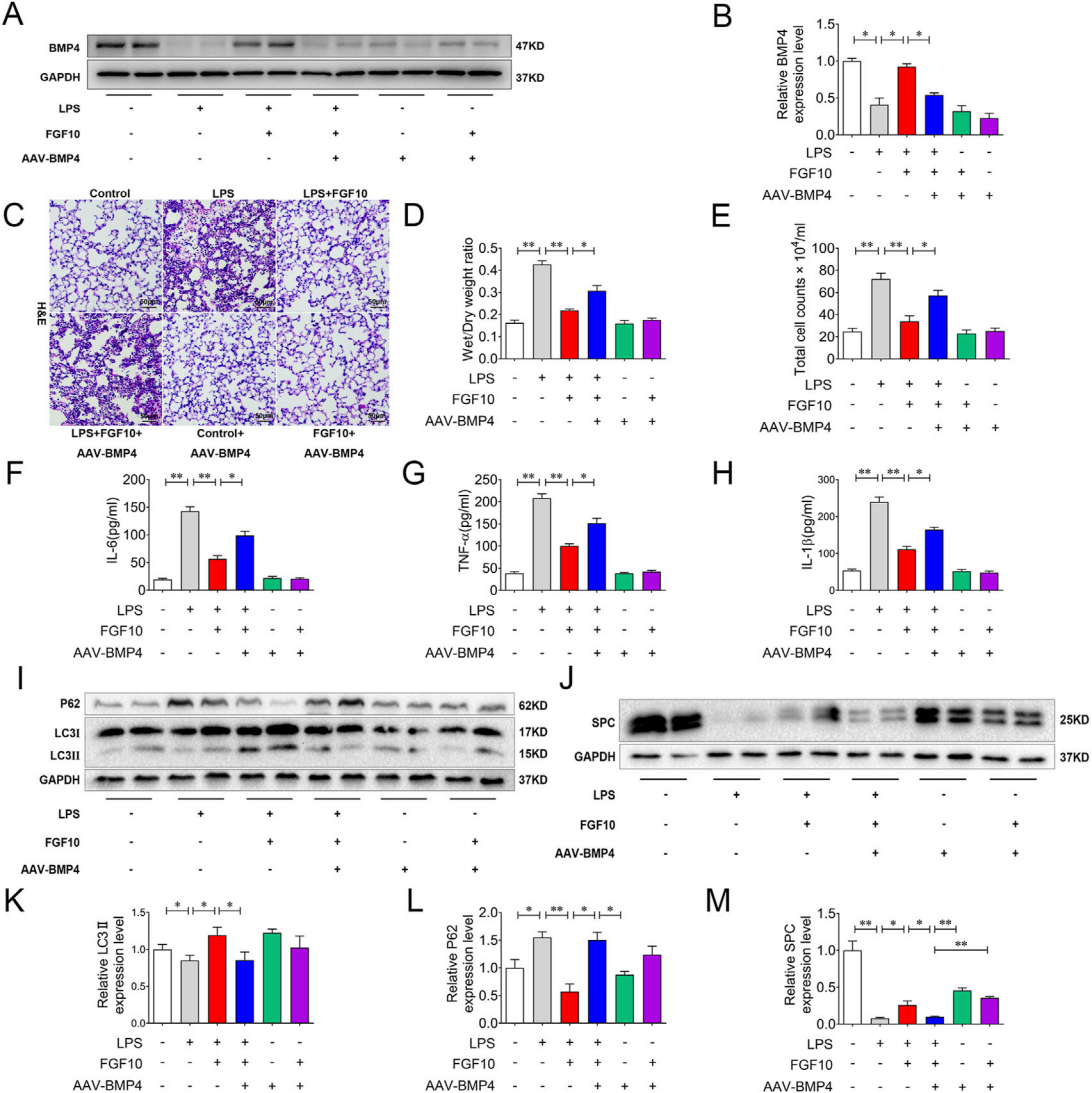
BMP4 knockdown weakens the protective effect of FGF10 on acute lung injury by autophagy. **(A)** The expression of BMP4 in mice was detected by Western blotting. **(B)** Quantitative analysis of BMP4 protein. **(C)** Pathological change in lung tissue was detected by H&E (200×). **(D)** Quantitative analysis of wet/dry weight ratio. **(E)** BAL fluid was obtained to differentially count total cells. **(F–H)** The expression of IL-6, TNF-α, and IL-1β in BALF fluid was analyzed by ELISA. **(I,J)** The expression of LC3, P62, and SPC in mice was detected by Western blotting. **(K–M)** Quantitative analysis of LC3, P62, and SPC protein. The data were presented as the mean ± SEM, n = 5. *p < 0.05, * *p < 0.01.

To confirm the role of BMP4 in the FGF10-protective effect in the LPS injury model, we injected AAV-BMP4 into the mice by tail veins to knock down BMP4. BMP4 knockdown mice and control mice were treated with LPS and FGF10. Western blotting analysis showed that the expression of BMP4 was significantly decreased and the promotion of BMP4 by FGF10 was also weakened in BMP4 knockdown mice (Figures 5A,B). A model of BMP4 knockdown mice was established successfully. Accordingly, we examined for histological changes in H&E-stained lung tissue of mice and found that compared with the control group, the lung morphology of BMP4 knockdown mice showed no significant change, but compared with the FGF10 + LPS group, BMP4 knockdown mice weakened the protective effect of FGF10 in acute lung injury, increased alveolar injury, structural damage, and marked infiltration of inflammatory cells (Figure 5C). Compared with the FGF10 + LPS group, there was a significant increase in the wet/dry ratio of lung tissue and the levels of total protein, IL-6, TNF-α, and IL-1β in the alveolar lavage fluid of BMP4 knockdown mice treated with FGF10 + LPS (Figures 5D–H). It has been suggested that FGF10 improvement of ALI could be related to increased protein BMP4 (Zhao et al., 2013; Deng et al., 2018; Li et al., 2020). We have previously demonstrated that FGF10 promotes autophagy to improve ALI; therefore, we suspect that there is a link between BMP4 and autophagy. To test this hypothesis, we examined the changes in autophagy, specifically in the lung tissue. Interestingly, Western blotting results showed that compared with the FGF10 + LPS group, BMP4 knockdown significantly decreased the ratio of LC3II/LC3I and increased the expression of P62 in the FGF10 + LPS group (Figures 5I,K,L). Meanwhile, FGF10 induced an increase in SPC, which was also decreased by BMP4 knockdown (Figures 5J,M). These results showed that BMP4 knockdown mice weakened the protective effect of FGF10, which could be related to a decrease in autophagy and the regeneration of alveolar epithelial cells.

### BMP4 inhibition attenuates the protective effect of FGF10 on LPS-AECs injury by downregulation of autophagy

**FIGURE 6:**
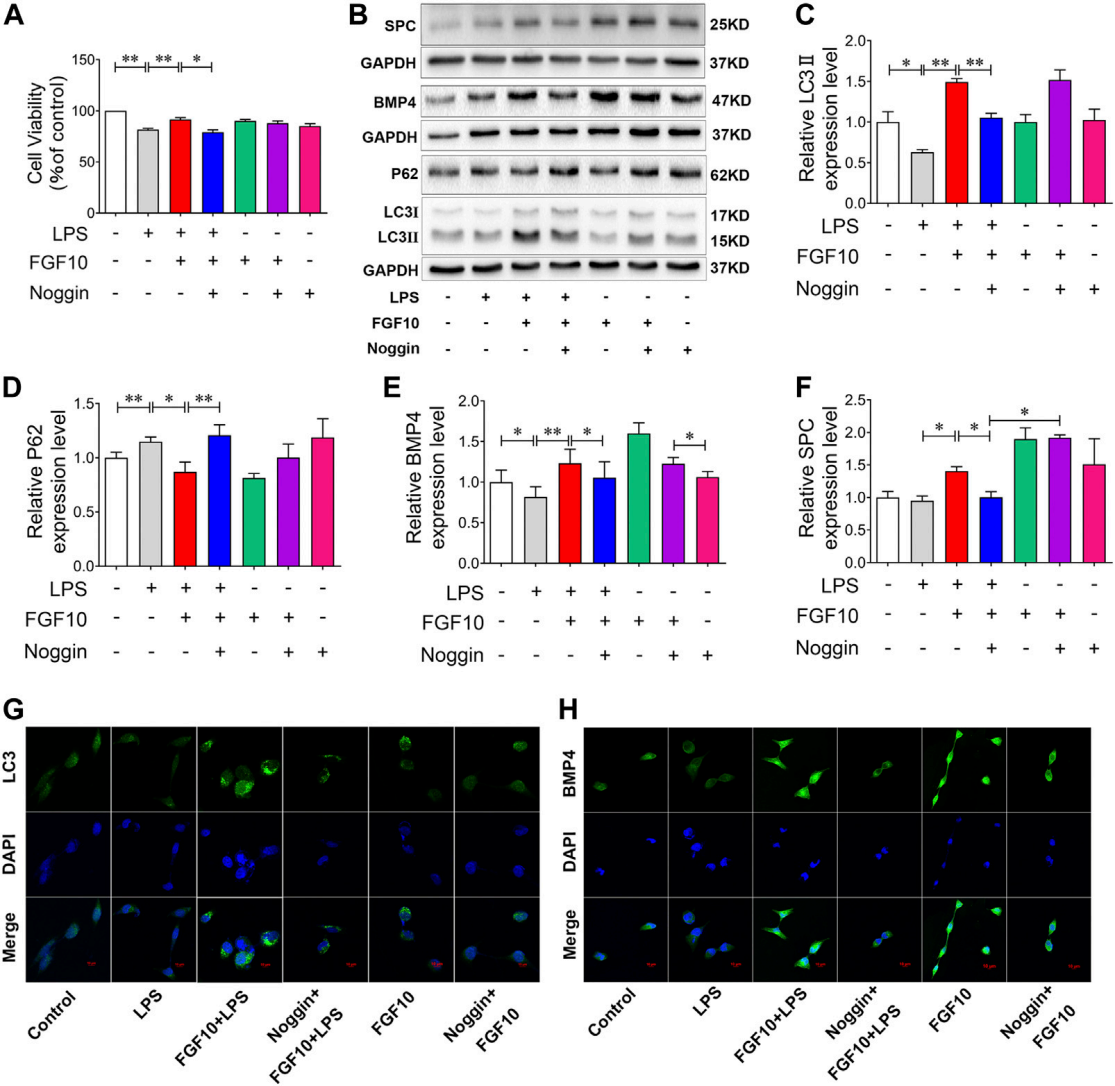
Inhibition of BMP4 decreases the protective role of FGF10 on AECs *via* autophagy. **(A)** After exposure to 200 ng/ml of Noggin for 0.5 h and 10 ng/ml of FGF10 for 1 h, the viability of AECs with 10 μg/ml LPS administration was detected by CCK-8. **(B)** The protein expression of LC3, P62, BMP4, and SPC was examined by Western blotting. **(C–F)** Quantitative analyses of LC3, P62, BMP4, and SPC protein. **(G,H)** The expression of LC3 (green) and BMP4 (green) of AECs were analyzed by immunostaining (magnification ×400). The data were presented as the mean ± SEM, n = 3. *p < 0.05, * *p < 0.01.

To further verify the role of BMP4 in FGF10-activated autophagy, we added Noggin, which can inhibit the expression of BMP4, to AECs for 30 min in advance to observe the protective effect of FGF10. CCK-8 assay results showed that compared with the control group, Noggin had no effect on cell viability, but it could attenuate the protective effect of FGF10 on AECs after LPS injury (Figure 6A). Furthermore, we examined the effect of BMP4 on FGF10-activated autophagy. Western blotting showed that compared with the LPS group, treatment with FGF10 markedly increased BMP4, LC3II/LC3I ratio, and decreased P62 levels, but this effect was reversed by Noggin in AECs (Figures 6B–E). Additionally, we tested the expression of SPC. Western blotting showed that FGF10 significantly increased the expression of SPC in LPS-treated cells, which was reduced by Noggin treatment (Figures 6B, F). Subsequently, the expression of LC3 and BMP4 was detected using immunofluorescence. The results showed that, compared with the FGF10 + LPS group, the expression of LC3 and BMP4 significantly decreased in cells with the addition of Noggin (Figure 6G, H). These results were consistent with those of the *in vivo* experiments. These data demonstrate that FGF10 promotes the regeneration of alveolar type II cells and improves acute lung injury *via* BMP4/autophagy signaling.

### FGF10-activated autophagy protects AECs injury induced by LPS

**FIGURE 7:**
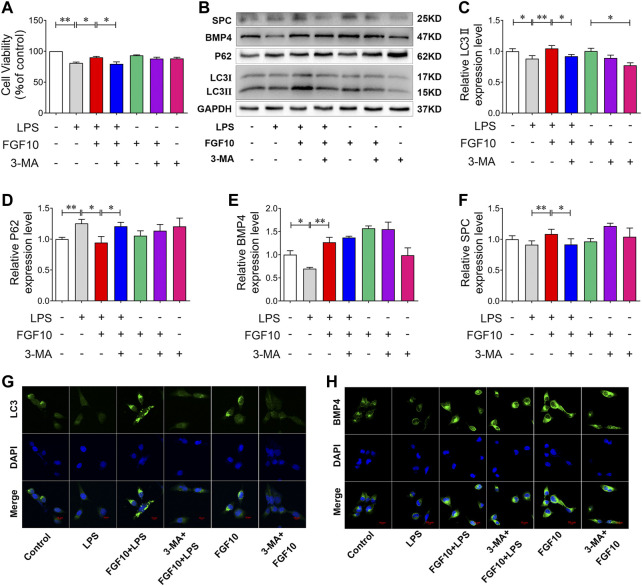
Inhibition of autophagy exacerbates cytotoxicity induced by LPS in AECs. **(A)** After exposure to 2.5 mM of 3-MA for 2 h and 10 ng/ml of FGF10 for 1 h, the viability of AECs with the administration of 10 μg/ml of LPS was detected by CCK-8. **(B)** The protein expression of LC3, P62, BMP4, and SPC was examined by Western blotting. **(C–F)** Quantitative analyses of LC3, P62, BMP4, and SPC protein. **(G,H)** The expression of LC3 (green) and BMP4 (green) of AECs was analyzed by immunostaining (magnification ×400). The data are presented as the mean ± SEM, n = 3. *p < 0.05, * *p < 0.01.

To further confirm the role of FGF10-promoted autophagy in LPS-induced ALI, we added a non-specific inhibitor of PI3-kinase, 3-MA, to further observe the protective effect of FGF10. CCK-8 results showed that 3-MA had no effect on cell viability, but attenuated the protective effect of FGF10 in LPS-induced AEC injury (Figure 7A). Furthermore, autophagy was analyzed by Western blotting, and the results showed that compared with the FGF10 + LPS group, 3-MA alone significantly inhibited the ratio of LC3II/LC3I, but increased the expression of P62 in FGF10 protected ACEs injury induced by LPS (Figures 7B–D). However, 3-MA administration did not affect the increase in BMP4 expression induced by FGF10 (Figure 7B, E), indicating that autophagy acts as a downstream signal of BMP4 in the protection FGF10. Additionally, Western blotting results showed that 3-MA also decreased the expression of SPC in the FGF10 + LPS group cells (Figure 7F). These results indicated that 3-MA decreased the ability of FGF10 to promote regeneration of alveolar type II cells. To confirm the change in autophagy and BMP4 in ACEs treated with different factors, the expression of LC3 and BMP4 was detected by immunofluorescence. These results are similar to the results of Western blotting. 3-MA decreased LC3 fluorescence density and did not affect the expression of BMP4 in cells treated with FGF10 and LPS (Figure 7G,H). These results suggest that FGF10 protects against LPS-induced lung injury *via* the promotion of autophagy.

## Discussion

As a common and serious pulmonary disease, ALI has a high mortality rate (30%–40%) without targeted therapy. Previous studies have shown that FGF10 can improve LPS-induced lung injury (Chen et al., 2020; Liu et al., 2020). In our study, we examined the expression of FGF10 and its receptor FGFR2b in LPS-induced ALI, specifically in mice. The results showed that the expression of FGF10 was significantly increased by LPS treatment, while FGFR2b expression did not change significantly. The results showed that FGF10 played an important role in LPS-induced ALI, but the specific role and mechanism of FGF10 in ALI were not clear. To further clarify the role of FGF10 in lung injury, we used FGF10 intervention and found that FGF10 can reduce lung injury and reduce the expression of inflammatory factors. These results confirm that an increased FGF10 expression improves LPS-induced acute lung injury.

Alveolar epithelial cell injury is an important factor in ALI. Uncontrolled inflammation resulting from ALI leads to alveolar epithelial cell damage, including the disruption of intercellular connections between alveolar epithelial cells, damage to the plasma membrane, cytoskeletal reorganization, and reduction or disappearance of surfactants, thus leading to pulmonary edema, atelectasis, and finally, acute hypoxic respiratory failure or dysfunction (Liang et al., 2016). Studies on the repair mechanism of the lung epithelium have found that, after lung injury, the recovery of intact epithelium is also crucial for the recovery of lung homeostasis. Injury leads to the release of some factors that contribute to the repair mechanism, including pre-epidermal growth and fibroblast growth factor family members (TGF-α, FGF, HGF), McP-1, interleukin (IL-1, IL-2, IL-4, IL-13), and PGE2 (Crosby and Waters, 2010). FGF10 has been shown to improve ALI by inducing the transformation of bronchial epithelial stem cells into alveolar type II cells through the FGF10-FGFR2B signaling pathway (Yuan et al., 2019). Our results also showed that FGF10 can promote the regeneration of alveolar epithelial type II cells, further demonstrating the ameliorative effect of FGF10 on ALI.

The role of autophagy has long been controversial. Moderate activation of autophagy may both be beneficial in some lung diseases and harmful to the body in some cases (Liu et al., 2021; Yang et al., 2021). Studies have shown that the autophagy-activating drug rapamycin can inhibit apoptosis in alveolar epithelial cells induced by silicon dioxide nanoparticles (Zhao et al., 2019). Inhibition of mTOR to enhance autophagy can attenuate LPS-induced lung injury and pulmonary barrier function (Qin et al., 2020). In our study, we evaluated autophagy by detecting LC3 and P62 in the lungs of mice and human alveolar epithelial cells. The results showed a decreased LC3II/LC3I ratio and increased P62 expression in the LPS group; however, this was reversed by FGF10 treatment, indicating that FGF10 improves ALI by activating autophagy.

BMP4, a bone morphogenetic protein, exists in many cell types (Li et al., 2019). Some studies showed that during lung development, Wnt/β-catenin signaling stimulates the production of FGF10, which further induces the expression of BMP4 (Fujimori et al., 2010; McCormack et al., 2013; Zhang et al., 2015). However, the role of BMP4 in lung diseases and the regulatory effect of FGF10 on BMP4 are not fully understood. It has been shown that exogenous BMP4 can attenuate the upregulation of inflammatory mediators in LPS-induced airway epithelial cells and peritoneal macrophages (Li et al., 2014). BMP4 regulates airway regeneration after acute injury, including the downregulation of E-cadherin and induction of epithelial cells migration (Masterson et al., 2011). Meanwhile, During lung development, as a downstream signaling molecule of FGF10, BMP4 is also regulated by FGF10 (Yuan et al., 2018). Our study found that BMP4 expression decreased significantly in mice and alveolar epithelial cells treated with LPS. The expression of BMP4 was significantly increased by the exogenous administration of FGF10. BMP4 also plays an important role in FGF10-protected ALI. To further prove the protective effect of BMP4 in FGF10 on LPS-induced lung injury, we used BMP4 knockdown mice and the BMP4 inhibitor, Noggin. Inhibition of BMP4 markedly decreased FGF10-improved abnormal changes in lung histomorphology and infiltration of inflammatory cells, as revealed by HE staining. FGF10 decreased the release of inflammatory mediators IL-6, TNF-α, and IL-1β, which were also inhibited by inhibition of BMP4. Meanwhile, Western blotting results showed that inhibition of BMP4 significantly repressed the effect of FGF10 on autophagy and the expression of SPC. The results indicated that decreasing the expression of BMP4 reduced the activation of autophagy by FGF10 and prevented the regeneration of alveolar epithelial type II cells. These results suggest that the activation of autophagy by FGF10 may be related to an increase in BMP4 expression. This is consistent with the findings of Zhao et al. (2013) who demonstrated that BMP4 increases resistance to leukemia chemotherapy drugs by activating autophagy and subsequently inhibiting apoptosis. Additionally, Deng et al. (2018) found that BMP4 could activate autophagy to promote hepatoma cell proliferation . We found similar results in the current study, wherein the inhibition of BMP4 significantly decreased autophagy induced by FGF10 *in vivo* and *in vitro.* Therefore, this evidence shows that FGF10 improves ALI induced by LPS and is associated with the activation of BMP4/autophagy signaling.

In conclusion, FGF10 can improve LPS-induced acute lung injury by activating autophagy and promoting the regeneration of alveolar epithelial type II cells. The effect of FGF10 on autophagy may be related to an increase in BMP4 expression.

## Data Availability

The original contributions presented in the study are included in the article/supplementary material, further inquiries can be directed to the corresponding authors.
